# A Review on Biomechanics of Anterior Cruciate Ligament and Materials for Reconstruction

**DOI:** 10.1155/2018/4657824

**Published:** 2018-05-13

**Authors:** M. Marieswaran, Ishita Jain, Bhavuk Garg, Vijay Sharma, Dinesh Kalyanasundaram

**Affiliations:** ^1^Centre for Biomedical Engineering, Indian Institute of Technology Delhi, New Delhi, India; ^2^Department of Bioengineering, Indian Institute of Technology Kanpur, Kanpur, India; ^3^Department of Orthopaedics, All India Institute of Medical Sciences, New Delhi, India; ^4^Department of Orthopaedics, J P Narayana Trauma Centre, All India Institute of Medical Sciences, New Delhi, India; ^5^Department of Biomedical Engineering, All India Institute of Medical Sciences, New Delhi, India

## Abstract

The anterior cruciate ligament is one of the six ligaments in the human knee joint that provides stability during articulations. It is relatively prone to acute and chronic injuries as compared to other ligaments. Repair and self-healing of an injured anterior cruciate ligament are time-consuming processes. For personnel resuming an active sports life, surgical repair or replacement is essential. Untreated anterior cruciate ligament tear results frequently in osteoarthritis. Therefore, understanding of the biomechanics of injury and properties of the native ligament is crucial. An abridged summary of the prominent literature with a focus on key topics on kinematics and kinetics of the knee joint and various loads acting on the anterior cruciate ligament as a function of flexion angle is presented here with an emphasis on the gaps. Briefly, we also review mechanical characterization composition and anatomy of the anterior cruciate ligament as well as graft materials used for replacement/reconstruction surgeries. The key conclusions of this review are as follows: (a) the highest shear forces on the anterior cruciate ligament occur during hyperextension/low flexion angles of the knee joint; (b) the characterization of the anterior cruciate ligament at variable strain rates is critical to model a viscoelastic behavior; however, studies on human anterior cruciate ligament on variable strain rates are yet to be reported; (c) a significant disparity on maximum stress/strain pattern of the anterior cruciate ligament was observed in the earlier works; (d) nearly all synthetic grafts have been recalled from the market; and (e) bridge-enhanced repair developed by Murray is a promising technique for anterior cruciate ligament reconstruction, currently in clinical trials. It is important to note that full extension of the knee is not feasible in the case of most animals and hence the loading pattern of human ACL is different from animal models. Many of the published reviews on the ACL focus largely on animal ACL than human ACL. Further, this review article summarizes the issues with autografts and synthetic grafts used so far. Autografts (patellar tendon and hamstring tendon) remains the gold standard as nearly all synthetic grafts introduced for clinical use have been withdrawn from the market. The mechanical strength during the ligamentization of autografts is also highlighted in this work.

## 1. Introduction

**Figure 1:**
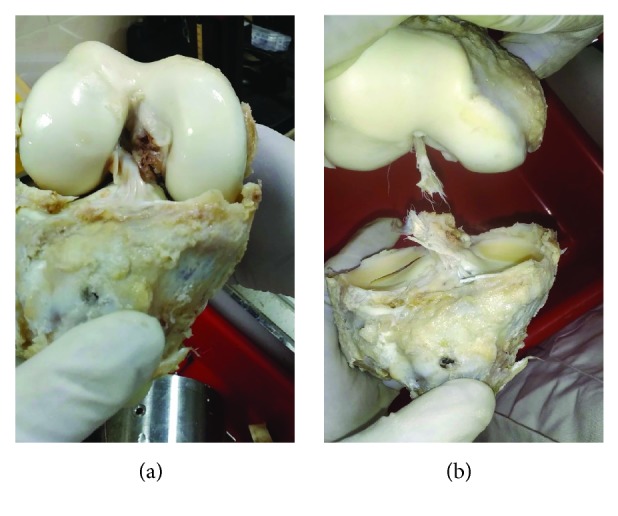
(a) ACL of a cadaver knee shown connecting the femur to the tibia. (b) A torn ACL.

The human knee joint is a complex joint and one of the important load-bearing joints of the body. The knee joint has two articulations: one between the tibia and the femur through menisci (tibiofemoral joint) and the other between the patella and the femur (patella-femoral joint). The anterior cruciate ligament (ACL) is one of the six ligaments that provide stability during the articulations. The ACL primarily restricts anterior sliding of the tibia over the femur thereby preventing hyperextension of the knee joint [1]. The ACL ranges from 25 to 35 mm in length, approximately 10 mm in breadth, and 4 to 10 mm in width. It is roughly triangular in cross-section and tapers along its length from both the ends up to the midsection; that is, the ACL has a higher cross-section at the bony interfaces and thinner at the midsection. Cruciate ligaments connect both the femur and the tibia in the central region and are unexposed for the most part of flexion and extension unlike medial and lateral collateral ligaments. The ACL is connected to the femur slightly posterior to the medial surface of the lateral condyle (LC) and to the tibia, at the anterior of the intercondylar region (ICR) as shown in Figure 1. Among various modes of injury, noncontact actions (during sports activities) are the significant cause of ACL injury [2]. During the noncontact action, the ACL is injured either partially or completely when the knee is flexed with the tibia rotating simultaneously in a lateral direction [2–6]. The ACL is stretched during flexion [7] and torqued during medial/lateral rotation. Injury of ACL impacts the active lifestyle of individuals retiring them into less cardiovascular activities [8], thereby affecting the overall health of the individual. Since the self-healing of injured ACL is literally absent [9] and given the severity of the injury (grade 2 or higher [10, 11]) or instability of the knee, surgical repair and/or replacement is required for the patient to return to an active sports life. In this work, we systematically review the following: anatomy of the ligaments, biomechanical forces under various kinematic positions, finite elemental analysis of ligaments, and ACL grafts.

### 1.1. Anatomy of Ligaments

Ligaments are tough, silvery white, dense connective tissue that connects bones directly or indirectly and stabilizes kinematic articulations. In direct insertion, the transition of the ligament to the bone occurs in the following sequence: ligament, fibrocartilage, mineralized fibrocartilage, and bone. In indirect insertion, the superficial fibers are attached to the periosteum and deep fibers are directly attached to the bone [12]. Ligament tissues are tough but somewhat pliable. Ligaments consist of fibroblast cells and the extracellular matrix (ECM). Fibroblasts are immature cells that have retained the ability to divide. These cells are large, branched, and flat and secrete (a) collagen fibers and (b) ground substance that constitutes the ECM. Fibroblast cells can migrate through the ECM. Collagen fibers are regularly arranged bundles of fibers in a parallel pattern. This arrangement provides mechanical resistance to pull force along the axis of fibers [1]. There are approximately 28 types of collagen fibers [13]. Of these, type I and III are the most abundant in ligaments [14]. Every fibrillar collagen molecule has three chains of polypeptides known as *α* chains. Each collagen type has either similar or dissimilar *α* chains; that is, collagen molecules can be either homotrimeric or heterotrimeric. Type I collagen has two different *α* chains out of the three chains, type II collagen has three identical *α* chains, and type III collagen has three different *α* chains [13]. Type I collagen is the most abundant fiber followed by type III. The two types of collagen are usually found together in the tendons, blood vessels, and so on. The two types of collagen may form either individual fibrils or one bundled fibril [15].

**Figure 2:**
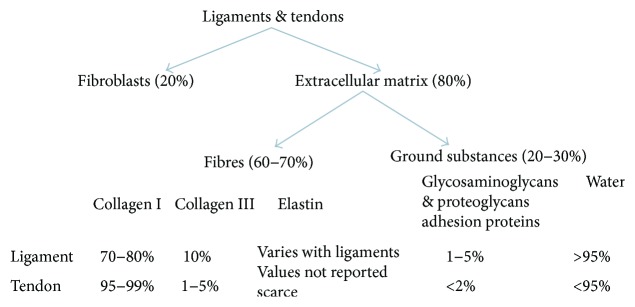
Composition of ligaments and tendons.

Ground substance constitutes the other part of ECM. Ground substance forms the matrix and has multiple functions such as (i) supporting and binding cells to one another as well as with the matrix, (ii) storing water, and (iii) serving as a medium/platform for the exchange of materials between cells and blood. Ground substance controls the overall metabolic activity of the tissue [1]. It also controls the process of changing the shape of the tissue. Ground substance is primarily composed of polysaccharides (also known as glycosaminoglycans (GAGs)) and proteins like elastin and so on. GAGs trap water, and this, in turn, gives the jelly-like appearance. GAGs are mostly associated with a protein core to form proteoglycans. GAGs are bound like bristles of bottlebrush to the protein core [16]. The ground substance also includes few adhesion proteins that play a major role in the linking of components of the ground substance to one another and to the surface of the cells. Integrins (family of cell surface proteins) play a major role in maintaining the framework between cell cytoskeleton and matrix [17]. Fibronectin is one such protein that binds the collagen fibers with the ground substance. Hyaluronic acid (polysaccharide present in ligaments) is viscous and slippery, found in the knee joint capsule. It lubricates knee joints and helps in cell binding. The composition of ligaments is briefed in Figure 2. As tendons are quite close to ligaments in terms of mechanical and biochemical characteristics, the composition of tendons is also provided in Figure 2 [14, 16].

**Figure 3:**
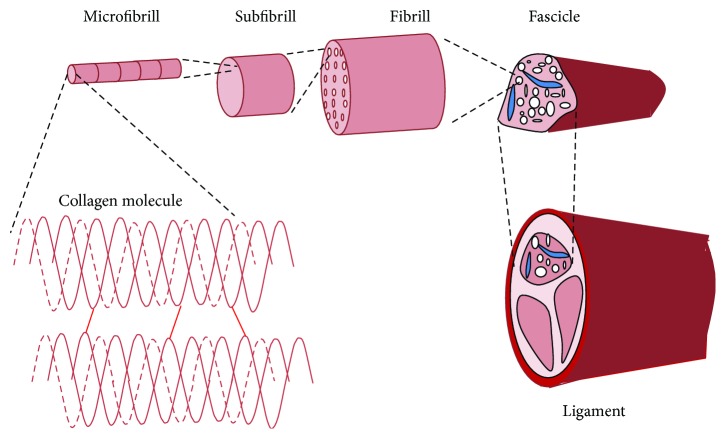
Schematic showing the hierarchy involved in the ligament [18].

The microstructure of the ligament is hierarchical in nature and is shown in Figure 3 [18]. The ligament is composed of bundled fascicles that are 50 to 300 *μ*m in diameter. The fibers have a multimodal distribution of diameter, and the diameter of fibers varies along the length. It includes both small and large fibers (10 to 500 nm) [19], and the fibers are packed tightly with smaller fibers squeezed in the gaps unoccupied by large fibers. Fascicles are formed from collagen fibrils (50 to 500 nm), and fibroblasts are aligned along the long axis of the ligament. Collagen fibrils display a wavy or sinusoidal arrangement know as the crimp pattern. The crimp can be observed in histology and scanning electron microscopy images along the longitudinal section. Collagen fibrils and fascicles exhibit the crimp pattern at every 67 nm and 45 *μ*m, respectively [18]. The crimp pattern, at different levels of magnification, results in gradual stiffening under tensile loading. The mechanical behavior of ligament is attributed to the crimp patterns. At the lower level, collagen fibrils are made up of microfibrils (3.5 nm).

#### 1.1.1. Slow Healing of ACL Injury

Nagineni et al. performed an *in vitro* cell culture study on ACL and MCL cell lines. Compared to MCL cells, ACL cells were observed to have low proliferation and migration potentials in response to injury [20]. Further, migration of fibroblast may be affected because of the abundance of fibronectin in the ACL and PCL when compared to the MCL and patellar tendon [21].

Silvers and Mandelbaum classified injuries to the ligament into three types. The first class of injury (grade I) results from a tear of less than one-third of the fibers in the ligament and presents knee laxity less than 5 mm. The second class (grade II) includes injuries resulting from the failure of one-third to two-thirds of fibers present in the ligament with a knee laxity of 5–10 mm. Grade III injuries result from the tear of more than two-thirds of the fiber with a knee laxity of 10–15 mm. The loss of function and tenderness is noticeable in grade II and grade III injuries. Knee laxity is measured as the anterior tibial translation from procedures used for clinical diagnosis. The anterior drawer test and Lachman test are performed clinically to diagnose ACL failure [22].

## 2. Biomechanics of ACL

### 2.1. Biomechanical Properties of ACL

**Figure 4:**
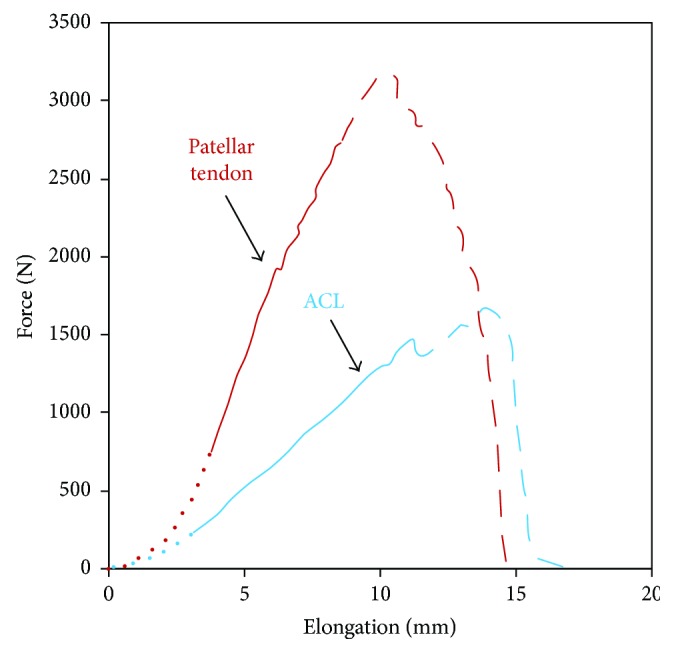
Tensile strength of ACL and the patellar tendon [33]. The dotted lines represent the toe region, continuous lines represent the linear region, and dashed/broken lines represent the yield region.

The stress-strain plot of ACL obtained under tensile loading shows a triphase graph, consisting of (i) the toe region, (ii) the linear region, and (iii) the yield region as shown in Figure 4. The crimp pattern in the collagen fibrils straightens out at low stresses, marking the toe region [23]. Resistance force gradually increases in the linear region with elastic deformation. The start of permanent deformation is marked by the yield region [24]. At this juncture, stress decreases due to the breakage of the collagen fibrils, eventually leading to ligament rupture. From the literature on cadaver studies, the ultimate tensile force of ACL varies between 600 and 2300 N (Table 1). Creep, stress-relaxation, and hysteresis with strain-rate dependency thereby indicating viscoelasticity of ligaments are also characteristics of ligaments. Ligaments can be studied by experimenting either isolated ACL or ACL with bone supports such as the femur-ACL-tibia complex (FATC). Research groups have preferred FATC samples over isolated ACL samples to avoid slippage during tensile testing. A study on variable strain rate behavior of isolated ACL has been carried out both in animals and in humans. Kennedy et al. studied the variable strain rate behavior of isolated human ACL. The authors reported that with an increase in strain rate, maximum load and strain to maximum load increased [25]. Isolated ACL studies on animals with the variable strain rate have not been reported.

Variable strain rate studies of FATC have been reported only on the rabbit [26], canine [27], and primates [28]. Pioletti et al. performed a stain rate study on bovine FATC at load levels much lower than failure load, that is, until 10% strain would occur [29]. The failure load and failure elongation have been increasing with the strain rate in few research groups' findings [26, 28] while failure load and failure elongation were observed to decrease in other groups' findings [27]. Hence, the behavior patterns of animal FATC were not consistent. In the case of human FATC, the variable strain rate behavior is yet to be reported. Sample preservation methods such as storage in saline (at room temperature), deep freezing, and embalming affect the fracture strength of ACL under loading [30]. There is no preservation method that can preserve samples (used for biomechanical testing) as well as fresh samples (i.e., samples available immediately after the death of human/animal or dissection of tissue from a live human/animal). Viidik and Lewin studied the effect of preservation methods on mechanical testing of rabbit ACL. According to the authors, the pattern of load-elongation curves obtained from both embalmed and fresh samples was similar but the magnitude of failure load was different. The team was able to compare fresh and embalmed samples of rabbit ACL. Moreover, dissection of unembalmed human cadavers is not advisable due to the possibility of infection.

**Figure 5:**
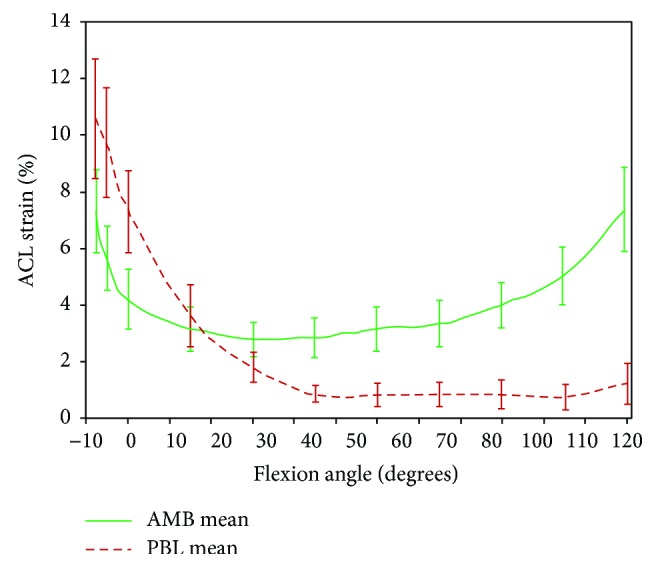
Average strain in AMB and PLB as a function of knee flexion angle; as shown in the figure, AMB is under tension during the extension at the knee joint and PLB is under tension during flexion [32].

Microscopically, the ACL is made up of two bundles, namely, anteromedial bundle (AMB) that is taut during flexion of the knee and posterolateral bundle (PLB) which is taut during the extension of the knee [31]. In other words, some portion of the ligament is under tension always.  Figure 5 shows the strain in the bundles as a function of the knee flexion angle [32]. In most surgical repairs, AMB is worked upon [7].

### 2.2. Kinematics and Kinetics of the Knee Joint during Level Walking and Stair Climbing

**Figure 6:**
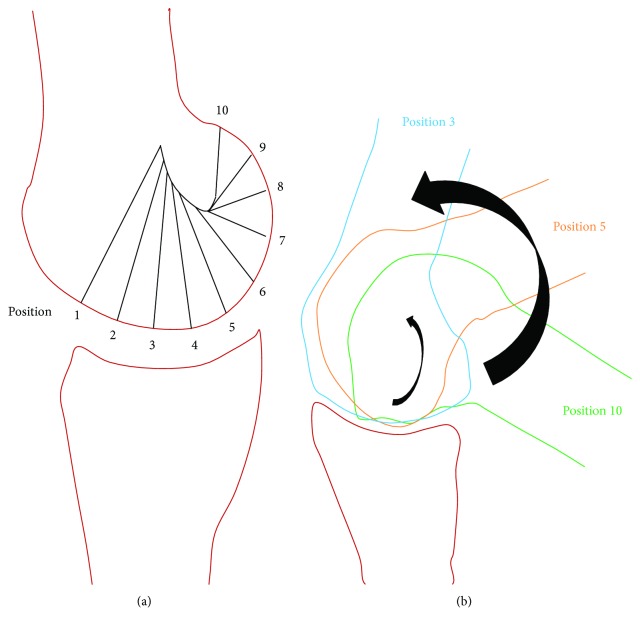
(a) Illustrating the change in the center of rotation (CoR) of the femur over the tibia (positions 1 to 10) (dotted lines indicate radii of rotation). (b) Arbitrarily selected anatomical positions during rotation.

Level walking and stair climbing involve flexion and extension at the knee joint. Quadriceps and hamstring is the antagonistic pair of muscles that aids in flexion and extension at the knee joint [38]. Flexion and extension of the knee joint include both the rotation of the tibia (with respect to the femur) and the translation of the femur over the tibia (forward/backward). Level walking involves up to 30° flexion at the knee joint. In the case of stair climbing, the knee flexion angle varies from 60° to 135°, depending on the height of each stair. The center of rotation (CoR) of the knee joint varies with respect to the angle of flexion. For the first 30° of flexion (i.e., positions 1 to 4 on Figure 6(a)), femoral condyle undergoes minimal anterior translation. Between 30° and 135°, the femoral condyle undergoes larger anterior translation.

**Figure 7:**
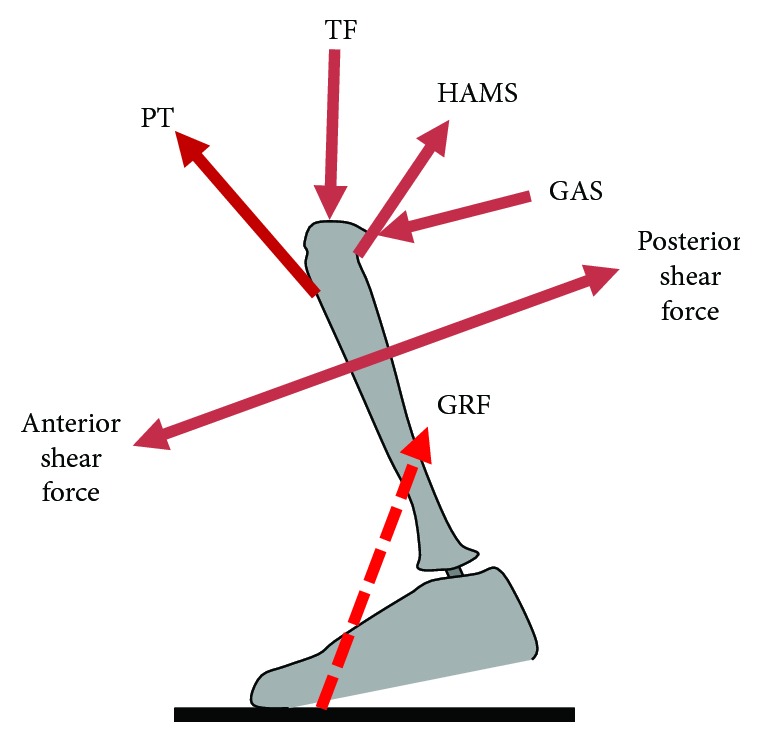
Forces acting at the knee joint [41]. TF: Tibiofemoral joint force; PT: patellar tendon force; HAMS: hamstring muscle force; GAS: gastrocnemius muscle force; GRF: ground reaction force.

Several muscle forces such as hamstring muscle force (HAMS), gastrocnemius muscle force (GAS), patellar tendon/quadriceps muscle force (PT), and joint contact force tibiofemoral (TF) act during flexion and extension of the knee joint. Quadriceps contract eccentrically during knee flexion and concentrically during extension. On the other hand, hamstring muscles perform an inverse action and therefore the two muscles—quadriceps and hamstrings—are antagonists. Hamstring muscles are attached behind the knee and therefore apply a posterior shear force on the tibia. The shear force due to the patellar tendon (high quadriceps force) has the largest share in determining the total shear force and occurs during the contralateral toe off (CTO). While walking, ground reaction force (GRF) occurs in addition to the above list of forces (Figure 7). GRF always applies a posterior shear force as the line of action of the resultant force pass behind the knee. The total shear force at the knee joint shall depend on the magnitude and direction of the individual forces. However, the maximum shear force is significantly dependent on the force exerted by the quadriceps muscle via the patellar tendon. Both anterior and posterior shear forces translate the femur over the tibia in the respective directions. These movements are restrained by the ACL.

#### 2.2.1. In Vivo Studies on Kinetics at the Knee Joint

Forces acting on the knee joint were measured by instrumented telemetrized implants *(in vivo)* by few research groups. The authors measured the axial force along with two shear forces (perpendicular to the implant axis). In the coordinate system followed, forces measured along the *x*-axis and *y-*axis (sagittal and coronal plane) were known as the shear force and the forces measured along the *z*-axis is known as the axial force (axial plane). The authors also measured three momentum components [39, 40] using six semiconductor-based strain gauges. The shear forces were found to be less than 10% of the magnitude of the axial force. The largest shear forces are found in level walking, ascending stairs, and descending stairs as compared to sitting down, standing up, and knee-bending activities.

### 2.3. Forces Acting on ACL

#### 2.3.1. Cadaveric Study

**Figure 8:**
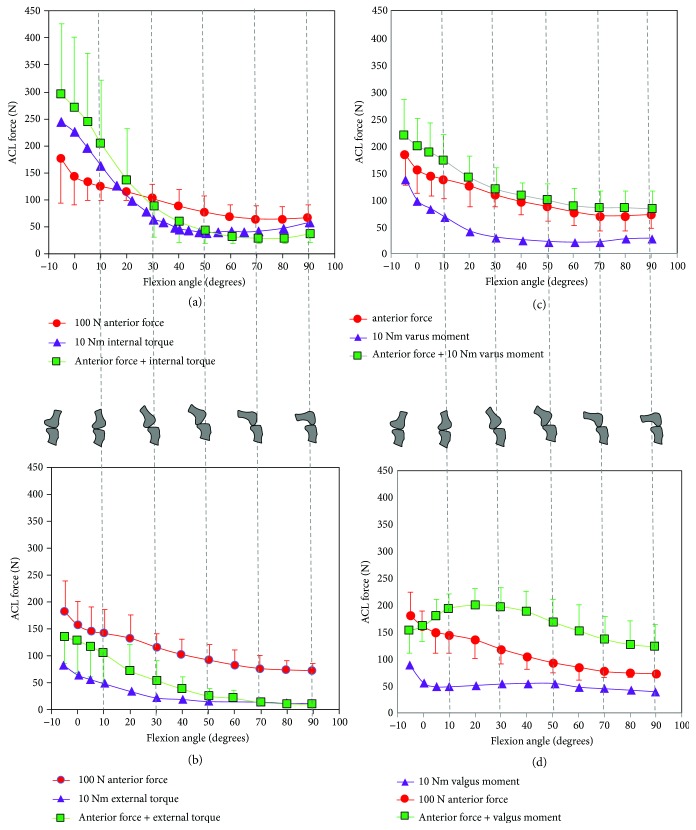
(a, b) The effect of combined loading on ACL force during knee flexion angles [42]. (c, d) The effect of combined loading on ACL force during knee flexion angles [42].

Markolf et al. analyzed forces acting on the ACL at various flexion angles under combinational loading (anterior force, internal/external torque, and varus/valgus motion) on an isolated cadaveric leg [42]. The force measurements were obtained via a load cell placed below the ACL insertion point on the tibia. Skin and other anatomical structures at the knee joint were left intact. This study was performed in a horizontal or supine position; that is, the loads due to the body weight of the cadaver and ground reaction force were absent. A mechanical arrangement was made to apply (a) either internal or external torque (10 Nm) and (b) either varus or valgus moment (10 Nm) at the knee joint. The ACL experienced the highest force for flexion angles less than 30° for all combinations of loads experimented. The highest ACL force of 300 N was observed at hyperextension (−5° of flexion) of the knee with 100 N anterior force and 10 Nm internal torque. ACL forces at various combinational loads are shown in Figure 8.

**Figure 9:**
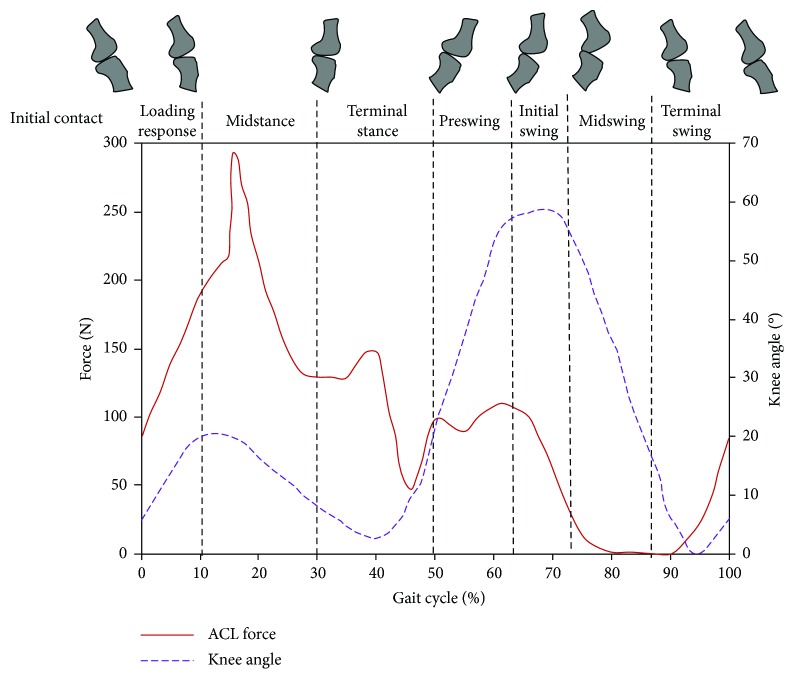
Forces acting on ACL during a simulated gait cycle along with changes in the knee angle during the gait cycle [41, 55].

Forces acting on the ACL were evaluated using simulated models during various phases of gait. The gait cycle during level walking can be divided into eight phases: (1) initial contact—heel strike (HS), (2) foot flat or loading response, (3) midstance or contralateral toe off (CTO), (4) terminal stance-heel off or contralateral heel strike (CHS), (5) preswing or toe off, (6) initial swing, (7) midswing, and (8) terminal swing. These phases are shown in Figure 9.

Morrison was the first researcher to calculate the force acting on the ACL through simulation. Nine males and 3 females were made to walk on the force plate and simultaneously imaged from both the front and the sides. The acceleration on each segment of the lower limb was calculated from each frame by the imaging of the gait. The ground reaction force and acceleration from the force plate and from images provided the total force acting on the knee. The maximum force acting on the ACL was calculated to be 156 N. The ACL was loaded during 5% to 25% of the gait cycle after heel strike [43]. The corresponding knee angle varies between 15° and 20°. In another study published by Collins, a sagittal plane model was used to estimate the forces acting on the ACL during the gait cycle. The effects of antagonistic and synergistic muscles were included in the dynamic analysis of level walking. About 900 N force was estimated to act on the ACL during the early stance phase [44].

#### 2.3.2. Computational Studies

Shelburne et al. [41] calculated and explained the pattern of loading on the ACL on the gait cycle of normal walking. Predicted ACL forces are shown Figure 9 as a function of the gait cycle as well as the knee angle. The authors benefited from a model developed and validated independently by Anderson and Pandy [45, 46]. Leg muscle forces, knee joint angles, and ground reaction forces were estimated from the “whole-body model” using the dynamic optimization theory. These predicted muscle and ground reaction forces from the “whole-body model” were used to estimate ACL forces via the musculoskeletal model of the lower limb. The study reports that the maximum loading of the ACL occurs during midstance. The corresponding knee angle varies between 15° and 20°. During the swing phase, the ACL was minimally loaded. The ligaments were assumed to be elastic in the above-cited mathematical models of Shelburne et al. [41].

### 2.4. Sex-Based Differences in Biomechanical Properties

Females are reported to suffer two- to seven-fold ACL injuries than their male counterparts of the same age [47, 48]. Excessive loads on ACLs per unit body weight are expected in females due to the lesser stiffness of the knee muscles [49]. In an extensive and detailed study by Hewett et al. [50], both male and female young athletes were assessed for a decade using coupled biomechanical-epidemiological approaches. The study found that the female players had four neuromuscular imbalances, namely, *ligament dominance*, *quadriceps dominance*, *leg dominance*, and *trunk dominance.* In the landing type of actions, the knees of female players tend to go in the valgus position. The posterior kinetic chain: the gluteals (maximus and medius), the hamstrings, the gastrocnemius, and the soleus, do not absorb a sufficient ground reaction force (GRF), forcing the joint and the ligament to absorb the high amounts of force. Despite the short duration of GRF occurrence, the damage is caused to the knee ligaments. This phenomenon is termed as *ligament dominance.* The second imbalance termed as *quadriceps dominance* relates to females using quadriceps muscles to stiffen the knee and stabilize the joint without the involvement of the posterior muscle chain which leads to the generation of an anterior shear force at the knee. The ACL that serves to check the anterior-posterior translation endures detrimental effects due to the anterior shear force. The third type of imbalance is *leg dominance* that relates to one-leg dominance in females that results in greater asymmetry between the lower limbs and the greater risk of future injury. The fourth type of imbalance is the *trunk dominance*. The imbalance relates to the inability to precisely control the trunk in a three-dimensional space. Given the fact that the females' center of mass (COM) is higher off the ground in comparison to the males' COM, the addition of trunk mass after maturation, without the muscles for control, amplifies the imbalance leading to higher lateral movement during sports activities. The distribution of mass in novel ways, higher COM, and lack of muscular control contribute to trunk imbalance [50]. In addition, the cross-sectional area, length, and volume of ACL are smaller in females than in males [51–54].

Maximum load at failure (1266 N (SD 527)), stiffness (198 N/mm (SD 88)), and modulus of elasticity (99 MPa (SD 50)) of ACL obtained from female cadavers were lower than that of male cadavers (1818 N (SD 699), 308 N/mm (SD 89), and 128 MPa (SD 5), resp.). As modulus of elasticity is independent of size, the above-reported differences in the value of modulus among genders indicate a compositional variation in the ACL. In a separate *in vitro* study on human ACL, estrogen was found to decrease the concentration of collagen which could answer the gender-based difference in mechanical properties of the ACL [56]. Hashemi et al. have reported that the ACL of females has lower fibril concentration and percentage of area occupied by collagen fibrils than the ACL of males [57].

## 3. Replacement Grafts

Structural and mechanical properties of the grafts prior to implantation, graft placement, revascularization, rehabilitation, and protection are the critical factors for the selection of the grafts.

### 3.1. Natural Grafts

**Figure 10:**
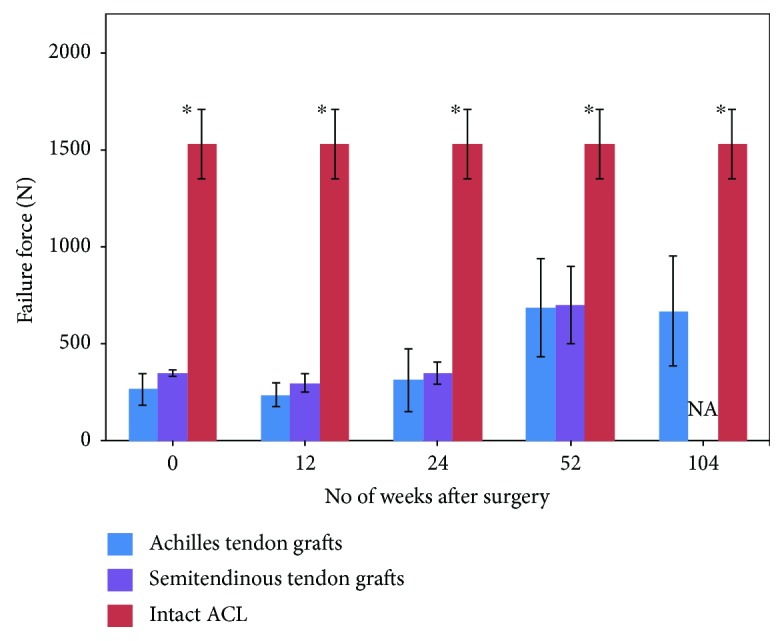
Comparison of failure force for autografts at various time points post ACL replacement surgery with intact ACL. ^∗^Significant difference (*p* < 0.05) between autografts and intact ACL for the corresponding time period [61, 62].

Natural grafts can be classified as (i) autografts, (ii) allografts, and (iii) xenografts. Grafts harvested from the patient's own tendon (partly) for the reconstruction of the torn ACL [10] are termed as autografts. Autografts reduce foreign body rejections, potential allergic reactions, and any disease transmission. Most common choices for autografts are bone-patellar-tendon-bone (B-PT-B), quadriceps tendon, and hamstring tendon (semitendinosus-gracilis). Autograft-based ACL surgical methods demand more surgery time as well as recovery time due to additional incision in the patient's body. Graft site morbidity may also have had a detrimental effect on the process. The width of the graft and availability of bony insertion points decide the success of the autograft for ACL replacement. Due to tissue necrosis after implantation, all autografts undergo weakening. Hence, the initial strength of the autografts at the time of harvesting should be sufficiently larger than native ACL to make up for the loss in strength arising due to tissue necrosis [33].  Figure 4 [33] illustrates the tensile strength of ACL and the patellar tendon (PT) to highlight the difference in a mechanical behavior. The difference in the composition of ground substance and fiber arrangement causes the tendon to stretch shorter and absorb higher load. However, due to shorter strain characteristics of PT autografts, there may be a mismatch in laxity in a PT-implanted knee as compared to the contralateral knee with natural ACL. Autografts (mostly patellar tendon or hamstring tendon) undergo the process of ligamentization over a period of 24 months after surgery. This activity is documented well in an animal model. In the few human studies available, the time required for complete ligamentization was observed to vary as reported by research groups [58, 59]. Histologically, the tendon graft has undergone changes towards ligament but collagen distribution still remains unchanged or minimally changed [60]. Weiler et al. evaluated the biomechanical properties of the Achilles tendon split graft in sheep over a period of two years. Maximum load to failure, stiffness, and tensile strength were observed to be significantly lesser than the intact ACL even after 104 weeks [61]. Kondo et al. performed a similar study for the semitendinosus tendon graft for one year in sheep and obtained similar results [62]. The failure force of the graft was significantly lower than the intact ACL after 52 weeks in spite of ligamentization. Only stiffness (i.e., slope of force versus deformation) of the graft was comparable to the intact ACL after 52 weeks. This trend suggests that the ligamentization process revives the graft histologically and not mechanically. The key findings of the studies by Weiler et al. and Kondo et al. are summarized in Figure 10. Failure force for two autografts for the ACL at various time points after surgery is compared with intact ACL.

Grafts obtained from human cadavers are termed as allografts [63]. B-PT-B, Achilles tendon, hamstring tendons, and anterior/posterior tibialis are the various options for allografts. Allograft-based replacement surgeries require reduced surgery time on the patient and therefore shorter recovery time. Donor site morbidity is eliminated in this procedure. On the other hand, it has an increased surgical cost. Availability of donor, donor medical history, and sterilization processes of allografts affect the quality of the graft for replacement. However, there are high chances of infection of certain diseases and rejection of the graft altogether. Also, the high temperature and pressure during sterilization process may alter the biomechanical properties. The third source of natural grafts is from other animal species such as porcine and bovine, and these grafts are collectively termed as xenografts. These grafts are similar to allografts but with a greater risk of transmission of diseases and foreign body rejection [64]. In a survey conducted among surgeons, the hamstring tendon (63%) was the first preferred choice of autograft followed by the patellar tendon graft (23%). The third choice is allografts (11%) [65]. Grafting patellar tendons constrain knee extension and induces pain and discomfort over a long period as compared to grafting hamstring tendon. Besides, the patellar tendon has excellent initial fixation and better bone-bone integration [65].

### 3.2. Synthetic Grafts

Silver, stainless steel, nylon, and silk strings, and so on are few materials experimented for synthetic ligaments. Studies involving these materials did not clear animal studies due to early rupture and unsatisfied results [66]. Synthetic grafts include augmentation devices and permanent replacement [67]. Augmentation devices provide initial protection to autografts until it matures and revascularization happens. Permanent replacement grafts include carbon fibers and polymer fibers (poly-tetrafluoroethylene, polyester etc.). Various extinct carbon fiber-based commercial grafts were “Proplast” [Vitex Inc., Houston, USA], “Polyflex” [Richard, Memphis, USA], and “Intergraft” [Osteonics Biomaterials, Livermore, CA, USA]. The products failed to make an impact due to high rupture rate, deposition of carbon particles in the liver tissues, and inflammatory response in the surrounding tissues [68]. Polymeric fiber-based grafts that have been withdrawn are poly-tetrafluoroethylene- (PTFE-) based “Gore-Tex” [W. L. Gore, Flagstaff, AR, USA, 1986 [69]], polyester-based “Leeds-Keio” [Neoligaments Ltd., Leeds, UK, 1982 [70]], polyester-based “Dacron” [Stryker Corp., 1989, Country [71]], polypropylene-based “Kennedy LAD” [St. Paul, MN, USA, [72]], and polyethylene terephthalate-based “LARS” [Surgical Implants and Devices, Arc-sur-Tille, France [73]]. Due to leaching effects, polymeric particles were found in the body, for example, Gore-Tex [74] which led to the withdrawal of these products from the market. The other reasons for the failure of the polymeric grafts were low biocompatibility, poor abrasion and torsion resistance causing higher rupture rates, and wear debris causing complications.

### 3.3. Engineered Biografts

Tissue engineering approaches include *in vitro* culture of neoligaments using biodegradable scaffolds seeded with cells and growth factors. The neoligaments are then used as graft material for ACL replacement. Most commonly used bioderived scaffold materials are collagen [75], silk [76], hyaluronic acid [77], chitosan [78], and alginate [79]. Synthetic materials have been used as scaffold material, and these include but not limited to poly-diaxonane [80], poly-glycolic-acid [81], poly-L-lactic acid [82], poly-lactic acid-co-glycolic acid [83], and poly-caprolactone [84]. Since ACL is a fibrous tissue, mostly fibers were used for ligament engineering. Freeman et al. combined braiding and twisting to design a scaffold for ACL tissue engineering [23]. Similarly, Chung et al. designed a scaffold having a hierarchical structure, using braiding and twisting of fibers [85]. Polymeric filaments were braided to produce fibers, which were further interwoven to form grafts. By applying different weaving techniques of fibers at different levels of magnification, the hierarchy was achieved similar to native ACL. The twisting angle, braiding angle, and filament diameter combinations regulated the porosity of these grafts. Another design variant includes bony attachment to the ends of scaffold for bone-to-ACL integration. Laurencin et al. and Chung et al. reported scaffolds with three zones: two bony ends and one intra-articular region differing in porosity [85, 86].

In the recent times, bridge-enhanced ACL repair conceived and developed by Murray and her team is gaining popularity. The team uses polypropylene suture as a guide and collagen-platelet rich plasma (PRP) hydrogel as a bridge (containing cells and growth factors). Platelet in plasma was identified as a source to be a provisional scaffold and initiate ECM protein production by fibroblast. Lack of provisional scaffold in ACL injuries is the major reason for the absence of self-healing [87–90]. Bioenhanced ACL reconstruction was compared with ACL replacement surgeries on porcine. Animals operated with bridge-enhanced ACL repair had no osteoarthritis reported in one-year follow-up as compared to fresh frozen PT-based allografts [91]. Biomechanical properties, yield load and stiffness of ACL repaired by collagen-PRP hydrogel, were found to be similar to that of human ACL [92, 93]. FDA is granted, and human trials are on the way.

## 4. Conclusions

In this review, kinematics and kinetics of the knee joint during various activities such as level walking and stair climbing, as well as loads acting on the ACL during the above-mentioned activities (as a function of flexion angle), are discussed in detail. In addition, a brief review of the following is provided: (a) composition and anatomy of ACL, (b) natural grafts (autografts, allografts, and xenografts), (c) synthetic grafts, and (d) engineered biografts for replacement/reconstruction surgeries. The highlights of the reported studies are as follows:

- Mechanical behavior of cadaveric ACL/FATC
  - ACL behavioral pattern under quasistatic, torsional, and combinational loading in the reported literature does not overlap with each other. Various factors such as demography, sample preservation methods, differences in grippers used/gripping techniques, isolated ACL versus FATC samples, and varying strain rates could have contributed to the differences.
- Kinematics of the knee joint in level walking and stair climbing
  - Flexion and extension of the knee joint involve both the rotation of the tibia (with respect to the femur) and the translation of the femur over the tibia (forward/backward). Level walking involves up to 30° flexion at the knee joint while stair climbing involves flexion angle up to 135°, depending on the height of each stair.
  - The center of rotation (CoR) of the knee joint varies with respect to the angle of flexion. For the first 30° of flexion, femoral condyle undergoes minimal anterior translation. Between 30° and 135°, the femoral condyle undergoes larger anterior translation.
- Forces acting on ACL
  - The highest shear forces at ACL occur during hyperextension (−5° of flexion) of the knee joint. Combinational loading (i.e., shear loading along with torsional loading) is additive for flexion angles from −5° to 20° at the knee joint.
  - Forces applied on ACL during flexion/extension of the knee in combination with torque and moment were reported on isolated cadaveric knee experimented in the supine position. Hence, this test had excluded the effect of gravity and body weight. Actual force on live ACL tissue has not been quantified under various real-life loading and injury-causing situations. Due to practical limitations of predicting ACL forces on a live subject, these forces have been computed through analytical models.
  - Characterization of ACL at variable strain rates provides information on the viscoelastic nature of ACL. Though variable strain rate studies of FATC on primates, canines, rabbits, and so on, have been reported, similar studies on human FATC have not been reported. In addition to variable strain rate study, creep and stress relaxation studies of human ACL can help in the selection of material for the implant design.
- Graft material for ACL reconstruction
  - Natural grafts such as autografts are the most preferred option in the recent times. Most of the synthetic grafts have been recalled from the market. However, bridge-enhanced ACL repair developed by Murray et al. seems to be a promising technique for ACL reconstruction.

## Figures and Tables

**Table 1:** Human cadaveric studies on mechanical characterization of ACL and FATC.

Authors (year)	Number of subjects and age	Tensile force/strength, mean (SD) in Newton	Stiffness, mean (SD) in Newton/mm	Remarks/special considerations/comments
Kennedy et al. (1976)	10 isolated ACL samples Median age 62	626 (51)	—	Strain rate study on isolated ACL samples was performed. Failure load and strain increased as a function of strain rate [25]

Trent et al. (1976)	10 FATC samples Age between 29 and 55 years	633	141	FATC samples [34]

Noyes and Grood (1976)	6 FATC samples Age between 16 and 26 years	1730 (660)	182 (56)	The presented tensile behavior of FATC is considered gold standards [35]
	20 FATC samples Age between 48 and 86 years	734 (266)	129 (39)	Strength and stiffness of ligaments decrease with increase in age [35]

Woo et. al (1991)	54 FATC samples 3 age groups (22–35, 40–50, and 60–97) were studied	2160 (157) groups, 22–35 years 1503 (83) groups, 40–50 years 658 (129) groups, 60–97 years	242 (28) groups, 22–35 years 220 (24) groups, 40–50 years 180 (25) groups, 60–97 years	The effects of age and orientation direction (anatomical and tibial orientation) were studied. The younger population was observed to possess higher strength. Samples tested in anatomical direction had more tensile strength than those tested in tibial orientation [36]

Chandrashekar et al. (2006)	17 FATC (8 male FATC, 9 females) Mean age was 37 years	1818 (699) males, 1266 (527) females	308 (89) males, 199 (88) females	Male FATC samples were observed to fracture at higher loads than female FATC samples [37]

## References

[1] Tortora G. J., Derrickson B. H. (2009).

[2] Boden B. P., Dean G. S., Feagin J. A., Garrett W. E. (2000). Mechanisms of anterior cruciate ligament injury.

[3] Ferretti A., Papandrea P., Conteduca F., Mariani P. P. (1992). Knee ligament injuries in volleyball players.

[4] McNair P. J., Marshall R. N., Matheson J. A. (1990). Important features associated with acute anterior cruciate ligament injury.

[5] Olsen O. E., Myklebust G., Engebretsen L., Holme I., Bahr R. (2003). Relationship between floor type and risk of ACL injury in team handball.

[6] Olsen O.-E., Myklebust G., Engebretsen L., Bahr R. (2004). Injury mechanisms for anterior cruciate ligament injuries in team handball: a systematic video analysis.

[7] Levangie P. K., Norkin C. C. (2011).

[8] Roos E. M., Roos H. P., Lohmander L. S., Ekdahl C., Beynnon B. D. (1998). Knee Injury and Osteoarthritis Outcome Score (KOOS)—development of a self-administered outcome measure.

[9] Kiapour A. M., Murray M. M. (2014). Basic science of anterior cruciate ligament injury and repair.

[10] Daniel D. M., Akeson W. H., O’Connor J. J. (1990).

[11] Kessler M. A., Behrend H., Henz S., Stutz G., Rukavina A., Kuster M. S. (2008). Function, osteoarthritis and activity after ACL-rupture: 11 years follow-up results of conservative versus reconstructive treatment.

[12] Woo S. L.-Y., Inoue M., McGurk-Burleson E., Gomez M. A. (1987). Treatment of the medial collateral ligament injury. II: structure and function of canine knees in response to differing treatment regimens.

[13] Hulmes D. J. S., Fratzl P. (2008). Collagen diversity, synthesis and assembly.

[14] Robi K., Jakob N., Matevz K. (2013). The physiology of sports injuries and repair processes.

[15] Kühn K., Mayne R., Burgeson R. E. (1987). The Classical Collagens: Types I, II, and III.

[16] Culav E. M., Clark C. H., Merrilees M. J. (1999). Connective tissues: matrix composition and its relevance to physical therapy.

[17] Lane J. G., Amiel D., Gobbi A., Espregueira-Mendes J., Lane J., Karahan M. (2017). Ligament histology, composition, anatomy, injury, and healing mechanisms.

[18] Kastelic J., Galeski A., Baer E. (1978). The multicomposite structure of tendon.

[19] Ottani V., Raspanti M., Ruggeri A. (2001). Collagen structure and functional implications.

[20] Nagineni C. N., Amiel D., Green M. H., Berchuck M., Akeson W. H. (1992). Characterization of the intrinsic properties of the anterior cruciate and medial collateral ligament cells: an in vitro cell culture study.

[21] Amiel D., Foulk R. A., Harwood F. L., Akeson W. H. (1989). Quantitative assessment by competitive ELISA of fibronectin (Fn) in tendons and ligaments.

[22] Silvers H. J., Mandelbaum B. R. (2011). ACL injury prevention in the athlete.

[23] Freeman J. W., Woods M. D., Laurencin C. T. (2007). Tissue engineering of the anterior cruciate ligament using a braid–twist scaffold design.

[24] Silver F. H. (1994). Biomaterials, medical devices and tissue engineering: an integrated approach.

[25] Kennedy J. C., Hawkins R. J., Willis R. B., Danylchuck K. D. (1976). Tension studies of human knee ligaments. Yield point, ultimate failure, and disruption of the cruciate and Tibial collateral ligaments.

[26] Yamamoto S., Saito A., Nagasaka K. (2003). The strain rate dependence of mechanical properties of rabbit knee ligaments.

[27] de Lucas Resende J., Milton J., Saffar E. Experimental stress-strain curves for the knee cruciate ligaments.

[28] Noyes F. R., DeLucas J. L., Torvik P. J. (1974). Biomechanics of anterior cruciate ligament failure: an analysis of strain-rate sensitivity and mechanisms of failure in Primates.

[29] Pioletti D. P., Rakotomanana L. R., Leyvraz P. F. (1999). Strain rate effect on the mechanical behavior of the anterior cruciate ligament–bone complex.

[30] Viidik A., Lewin T. (2001). Changes in tensile strength characteristics and histology of rabbit ligaments induced by different modes of postmortal storage.

[31] Christel P., Franceschi J. P., Sbihi A., Colombet P., Djian P., Bellier G. (2005). Anatomic anterior cruciate ligament reconstruction: the French experience.

[32] Bach J. M., Hull M. L., Patterson H. A. (1997). Direct measurement of strain in the posterolateral bundle of the anterior cruciate ligament.

[33] Noyes F. R., Butler D. L., Grood E. S., Zernicke R. F., Hefzy M. S. (1984). Biomechanical analysis of human ligament grafts used in knee-ligament repairs and reconstructions.

[34] Trent P. S., Walker P. S., Wolf B. (1976). Ligament length patterns, strength, and rotational axes of the knee joint.

[35] Noyes F. R., Grood E. S. (1976). The strength of the anterior cruciate ligament in humans and rhesus monkeys.

[36] Woo S. L.-Y., Marcus Hollis J., Adams D. J., Lyon R. M., Takai S. (1991). Tensile properties of the human femur-anterior cruciate ligament-tibia complex.

[37] Chandrashekar N., Mansouri H., Slauterbeck J., Hashemi J. (2006). Sex-based differences in the tensile properties of the human anterior cruciate ligament.

[38] Collins J. J., O’Connor J. J. (1991). Muscle-ligament interactions at the knee during walking.

[39] Heinlein B., Kutzner I., Graichen F. (2009). ESB clinical biomechanics award 2008: complete data of total knee replacement loading for level walking and stair climbing measured in vivo with a follow-up of 6–10 months.

[40] Kutzner I., Heinlein B., Graichen F. (2010). Loading of the knee joint during activities of daily living measured *in vivo* in five subjects.

[41] Shelburne K. B., Pandy M. G., Anderson F. C., Torry M. R. (2004). Pattern of anterior cruciate ligament force in normal walking.

[42] Markolf K. L., Burchfield D. M., Shapiro M. M., Shepard M. F., Finerman G. A. M., Slauterbeck J. L. (1995). Combined knee loading states that generate high anterior cruciate ligament forces.

[43] Morrison J. B. (1970). The mechanics of the knee joint in relation to normal walking.

[44] Collins J. J. (1994). The redundant nature of locomotor optimization laws.

[45] Anderson F. C., Pandy M. G. (1999). A dynamic optimization solution for vertical jumping in three dimensions.

[46] Anderson F. C., Pandy M. G. (2001). Dynamic optimization of human walking.

[47] Arendt E., Dick R. (1995). Knee injury patterns among men and women in collegiate basketball and soccer.

[48] Griffin L. Y., Albohm M. J., Arendt E. A. (2006). Understanding and preventing noncontact anterior cruciate ligament injuries: a review of the Hunt Valley II meeting, January 2005.

[49] Granata K. P., Wilson S. E., Padua D. A. (2002). Gender differences in active musculoskeletal stiffness. Part I. Quantification in controlled measurements of knee joint dynamics.

[50] Hewett T. E., Ford K. R., Hoogenboom B. J., Myer G. D. (2010). Understanding and preventing Acl injuries: current biomechanical and epidemiologic considerations - update 2010.

[51] Anderson A. F., Dome D. C., Gautam S., Awh M. H., Rennirt G. W. (2001). Correlation of anthropometric measurements, strength, anterior cruciate ligament size, and intercondylar notch characteristics to sex differences in anterior cruciate ligament tear rates.

[52] Chandrashekar N., Slauterbeck J., Hashemi J. (2005). Sex-based differences in the anthropometric characteristics of the anterior cruciate ligament and its relation to intercondylar notch geometry: a cadaveric study.

[53] Hashemi J., Mansouri H., Chandrashekar N., Slauterbeck J. R., Hardy D. M., Beynnon B. D. (2011). Age, sex, body anthropometry, and ACL size predict the structural properties of the human anterior cruciate ligament.

[54] Muneta T., Takakuda K., Yamamoto H. (1997). Intercondylar notch width and its relation to the configuration and cross-sectional area of the anterior cruciate ligament. A cadaveric knee study.

[55] Kadaba M. P., Ramakrishnan H. K., Wootten M. E. (1990). Measurement of lower extremity kinematics during level walking.

[56] Liu S. H., al-Shaikh R., Panossian V. (1996). Primary immunolocalization of estrogen and progesterone target cells in the human anterior cruciate ligament.

[57] Hashemi J., Chandrashekar N., Mansouri H., Slauterbeck J. R., Hardy D. M. (2008). The human anterior cruciate ligament: sex differences in ultrastructure and correlation with biomechanical properties.

[58] Janssen R. P. A., Scheffler S. U. (2014). Intra-articular remodelling of hamstring tendon grafts after anterior cruciate ligament reconstruction.

[59] Pauzenberger L., Syré S., Schurz M. (2013). “Ligamentization” in hamstring tendon grafts after anterior cruciate ligament reconstruction: a systematic review of the literature and a glimpse into the future.

[60] Claes S., Verdonk P., Forsyth R., Bellemans J. (2011). The “ligamentization” process in anterior cruciate ligament reconstruction.

[61] Weiler A., Peters G., Unterhauser F. N., Su N. P. (2001). Biomechanical properties and vascularity of an anterior cruciate ligament graft can be predicted by contrast-enhanced magnetic resonance imaging. A two-year study in sheep.

[62] Kondo E., Yasuda K., Katsura T., Hayashi R., Kotani Y., Tohyama H. (2012). Biomechanical and histological evaluations of the doubled semitendinosus tendon autograft after anterior cruciate ligament reconstruction in sheep.

[63] Sun K., Tian S.-q., Zhang J.-h., Xia C.-s., Zhang C.-l., Yu T.-b. (2009). ACL reconstruction with BPTB autograft and irradiated fresh frozen allograft.

[64] Ellison A. E. (1979). Distal iliotibial-band transfer for anterolateral rotatory instability of the knee.

[65] Chechik O., Amar E., Khashan M., Lador R., Eyal G., Gold A. (2013). An international survey on anterior cruciate ligament reconstruction practices.

[66] Corner E. M. (1914). Notes of a case illustrative of an artificial anterior crucial ligament, demonstrating the action of that ligament.

[67] Legnani C., Ventura A., Terzaghi C., Borgo E., Albisetti W. (2010). Anterior cruciate ligament reconstruction with synthetic grafts. A review of literature.

[68] Rushton N., Dandy D. J., Naylor C. P. (1983). The clinical, arthroscopic and histological findings after replacement of the anterior cruciate ligament with carbon-fibre.

[69] Bolton W., Bruchman B. (1983). Mechanical and biological properties of the GORE-TEX expanded polytetrafluoroethylene (PTFE) prosthetic ligament.

[70] Fujikawa K., Kobayashi T., Sasazaki Y., Matsumoto H., Seedhom B. B. (2000). Anterior cruciate ligament reconstruction with the Leeds-Keio artificial ligament.

[71] Lukianov A. V., Richmond J. C., Barrett G. R., Gillquist J. (1989). A multicenter study on the results of anterior cruciate ligament reconstruction using a Dacron ligament prosthesis in “salvage” cases.

[72] McCarthy D. M., Tolin B. S., Schwenderman L., Friedman M. J., Woo S. L.-Y. (1993). Prosthetic replacement for the anterior cruciate ligament. The anterior cruciate ligament: current and future concepts.

[73] Lavoie P., Fletcher J., Duval N. (2000). Patient satisfaction needs as related to knee stability and objective findings after ACL reconstruction using the LARS artificial ligament.

[74] Indelicato P. A., Pascale M. S., Huegel M. O. (1989). Early experience with the GORE-TEX polytetrafluoroethylene anterior cruciate ligament prosthesis.

[75] Dunn M. G., Liesch J. B., Tiku M. L., Zawadsky J. P. (1995). Development of fibroblast-seeded ligament analogs for ACL reconstruction.

[76] Chen J., Altman G. H., Karageorgiou V. (2003). Human bone marrow stromal cell and ligament fibroblast responses on RGD-modified silk fibers.

[77] Cristino S., Grassi F., Toneguzzi S. (2005). Analysis of mesenchymal stem cells grown on a three-dimensional HYAFF 11®-based prototype ligament scaffold.

[78] Majima T., Irie T., Sawaguchi N. (2007). Chitosan-based hyaluronan hybrid polymer fibre scaffold for ligament and tendon tissue engineering.

[79] Majima T., Funakosi T., Iwasaki N. (2005). Alginate and chitosan polyion complex hybrid fibers for scaffolds in ligament and tendon tissue engineering.

[80] Buma P., Kok H. J., Blankevoort L., Kuijpers W., Huiskes R., Van Kampen A. (2004). Augmentation in anterior cruciate ligament reconstruction—a histological and biomechanical study on goats.

[81] Leong N. L., Petrigliano F. A., McAllister D. R. (2014). Current tissue engineering strategies in anterior cruciate ligament reconstruction.

[82] Lu H. H., Cooper J. A., Manuel S. (2005). Anterior cruciate ligament regeneration using braided biodegradable scaffolds: in vitro optimization studies.

[83] James R., Toti U. S., Laurencin C. T., Kumbar S. G. (2011). Electrospun nanofibrous scaffolds for engineering soft connective tissues.

[84] Peach M. S., Kumbar S. G., James R. (2012). Design and optimization of polyphosphazene functionalized fiber matrices for soft tissue regeneration.

[85] Chung E. J., Sugimoto M. J., Koh J. L., Ameer G. A. (2017). A biodegradable tri-component graft for anterior cruciate ligament reconstruction.

[86] Laurencin C. T., Freeman J. W. (2005). Ligament tissue engineering: an evolutionary materials science approach.

[87] Murray M. M., Spindler K. P., Ballard P., Welch T. P., Zurakowski D., Nanney L. B. (2007). Enhanced histologic repair in a central wound in the anterior cruciate ligament with a collagen–platelet-rich plasma scaffold.

[88] Murray M. M., Martin S. D., Martin T. L., Spector M. (2000). Histological changes in the human anterior cruciate ligament after rupture.

[89] Meaney Murray M., Spector M. (2001). The migration of cells from the ruptured human anterior cruciate ligament into collagen-glycosaminoglycan regeneration templates in vitro.

[90] Spindler K. P., Murray M. M., Devin C., Nanney L. B., Davidson J. M. (2006). The central ACL defect as a model for failure of intra-articular healing.

[91] Murray M. M., Fleming B. C. (2013). Use of a bioactive scaffold to stimulate anterior cruciate ligament healing also minimizes post-traumatic osteoarthritis after surgery.

[92] Joshi S. M., Mastrangelo A. N., Magarian E. M., Fleming B. C., Murray M. M. (2009). Collagen-platelet composite enhances biomechanical and histologic healing of the porcine anterior cruciate ligament.

[93] Murray M. M., Spindler K. P., Abreu E. (2007). Collagen-platelet rich plasma hydrogel enhances primary repair of the porcine anterior cruciate ligament.

